# The ILIA study: protocol for a randomized-controlled multicenter clinical trial on smartphone- and web-based relapse monitoring for patients with schizophrenia or schizoaffective disorder

**DOI:** 10.1007/s00406-025-02089-7

**Published:** 2025-08-19

**Authors:** Selina Hiller, Laura Emde, Denise Jais, Soňa Nevická Sikorová, Eduard Bakstein, Filip Španiel, Kateřina Urbanová, Eric Hahn, Marco Zierhut, Daniel Fürstenau, Markus Bühner, Lukas Junker, Isabel Maurus, Oliver Pogarell, Peter Falkai, Wolfgang Strube, Ingrid Bauer, Tobias Skuban-Eiseler, Josef Priller, Peter Brieger, Stephan Heres, Alkomiet Hasan, Kerem Böge, Stefan Leucht

**Affiliations:** 1 Technical University of Munich, TUM School of Medicine and Health, Department of Psychiatry and Psychotherapy, TUM University Hospital, Munich, Germany; 2 Department of Psychiatry and Neuroscience, Charité – Universitätsmedizin Berlin, Campus Benjamin Franklin, Berlin, Germany; 3 Mindpax.me, Prague, Czech Republic; 4 Center for first episodes of SMI, National Institute of Mental Health, Klecany, Czech Republic; 5 Analysis and interpretation of biomedical data, Faculty of Electrical Engineering, Prague, Czech Republic; 6 Department of Psychology, Faculty of Arts, Charles University (SMI NUDZ), Prague, Czech Republic; 7 DZPG (German Center for Mental Health), partner site Berlin, Berlin, Germany; 8 Berlin Institute of Health at Charité – Universitätsmedizin Berlin, BIH Biomedical Innovation Academy, BIH Charité Clinician Scientist Program, Berlin, Germany; 9 School of Business & Economics, Freie Universität Berlin, Berlin, Germany; 10 Institute of Medical Informatics, Charité – Universitätsmedizin BerlinFreie Universität Berlin and Humboldt Universität zu Berlin, Berlin, Germany; 11 Department of Psychology, LMU Munich, Munich, Germany; 12 Department for Psychiatry and Psychotherapy, LMU University Hospital, Munich, Germany; 13 Max-Planck-Institute for Psychiatry, Munich, Germany; 14 DZPG (German Center for Mental Health), partner site Munich/Augsburg, Munich, Germany; 15 Department of Psychiatry, Psychotherapy and Psychosomatics, Faculty of Medicine, University of Augsburg, Augsburg, Germany; 16 kbo-Isar-Amper-Klinikum Region München, München-Haar, Germany; 17 Institute of the History, Philosophy and Ethics of Medicine, Faculty of Medicine, Ulm University, Ulm, Germany; 18 Department for Psychology, Medical University Brandenburg, Neuruppin, Germany

**Keywords:** Digital monitoring, App, Shared-decision making, Schizophrenia, Outpatient treatment, Relapse

## Abstract

**Background:**

Despite the proven efficacy of antipsychotics in relapse prevention in schizophrenia and schizoaffective disorder, every third patient experiences a relapse within less than one year. Relapses can worsen psychosocial and treatment related outcomes and lead to substantial economic costs, primarily due to frequent and prolonged hospitalizations. The aim of this project is to evaluate a smartphone- and web-based digital solution for detecting early warning signs of schizophrenia and schizoaffective disorder to reduce relapses and subsequent hospitalizations.

**Methods:**

This randomized controlled trial compares the add-on use of a smartphone-based app for monitoring relapse warning signs in patients with schizophrenia and schizoaffective disorders (ICD-10 F20/F25) used within the routine psychiatric outpatient treatment against treatment as usual (TAU) without any further study-related intervention. Patients in the intervention group use the app for one year, fill in the weekly ten-item Early Warning Signs Questionnaire (EWSQ-10P) and obtain in-app feedback. Clinicians can access the symptom trajectory via a browser-accessible dashboard. If a threshold is exceeded in the inbuilt automatic algorithm, an alert is sent to both, the clinician and patient, enabling timely contact and, as part of a shared decision-making process, an optional adjustment of treatment decision. A total of 110 outpatients are recruited across eight study sites.

**Discussion:**

Continuous monitoring of early warning signs is expected to lead to behavioral changes and to decrease the necessity and duration of psychiatric hospital stays, thereby lowering healthcare costs. Additionally, the intervention could reduce symptom severity, alleviate medication adherence, shared decision-making, patient activation or quality of life. Qualitative data is collected to better understand patient needs and preferences regarding app usage and relapses. Insights gained from this study can be integrated into routine psychiatric care, improving the long-term treatment of patients with schizophrenia or schizoaffective disorder.

**Trial registration:**

German Clinical Trials Register (ID: DRKS00034991; registration date: 30.08.2024).

**Supplementary Information:**

The online version contains supplementary material available at 10.1007/s00406-025-02089-7.

## Introduction

Relapses occur frequently in patients with schizophrenia and schizoaffective disorder due to the chronic nature of these conditions [1–3]. Although a universally accepted definition of relapse in these disorders remains elusive, it is commonly characterized by psychiatric hospitalization or a measurable worsening of symptom severity [4–6]. Almost 40% of patients with schizophrenia and 28% of patients with an schizoaffective disorder require rehospitalization within twelve months after hospital discharge [7, 8]. Meta-analyses have shown that antipsychotics are more effective than placebo in relapse prevention. However, even with antipsychotic prophylaxis, 27% of patients experience a relapse within 10 months, whereas this figure is significantly higher at 61% under placebo [9, 10]. The evidence that relapses worsen disease progression and increase risk of treatment resistance is mixed [11, 12]. However, higher relapse rates are demonstrably associated with lower psychosocial outcomes such as unemployment, hopelessness or overall poorer quality of life [13], and patients often experience heightened anxiety about relapses [14, 15]. Above, associated costs are more than four times higher in relapsed schizophrenia patients than in non-relapsed patients [11]. Following the first two months after hospital discharge, the healthcare costs for patients with schizoaffective disorder are significantly higher than later on, mainly due to rehospitalizations and costs for medication [16]. Schizophrenia and schizoaffective disorder are both costly conditions, with the total systemic burden driven primarily by frequent and prolonged hospitalizations, as well as indirect costs such as unemployment and lost productivity [11, 17–20]. Developing strategies to reduce relapses is urgently needed and would substantially alleviate the disease burden for those affected, their families, and society.

Studies and guidelines unanimously emphasize the need to recognize early warning signs of relapse in schizophrenia, which have been shown to occur several weeks before a relapse [21–24]. They usually consist of non-specific symptoms such as sleep disturbances or fluctuations in affect, increasing disorganization of behavior or cognitive dysfunction [22, 25]. If early warning signs are recognized timely, not only by the clinician but also by patients and relatives, medication and other treatment measures can be adjusted, potentially reducing relapse rates [18, 26]. The ITAREPS program (Information Technology Aided Relapse Prevention Program in Schizophrenia) implemented a text message-based solution, where patients with schizophrenia weekly completed the 10-item Early Warning Signs Questionnaire (EWSQ-10P) on their phones [27]. A validated algorithm calculated the likelihood of relapse and alerted the clinician via text message when the risk was high, enabling timely intervention through medication adjustments or any other necessary measures. Using this approach, a randomized controlled trial showed that using the intervention in addition to routine treatment could reduce the hospitalizations by up to 60% compared to treatment as usual [28].

The present study aims to strengthen patient autonomy by actively involving patients with schizophrenia or schizoaffective disorder into a smartphone-based early warning system. Previous studies showed that shared-decision making tends to improve treatment satisfaction and potentially reduce rehospitalization rates [29, 30]. In contrast to the simplistic method of merely sending a text message to alert clinicians of an increased relapse risk, this study seeks to develop a digital solution that graphically displays the trajectory of early warning signs longitudinally on patientsˈ smartphones, making the information transparently accessible to both, patients and clinicians. We hypothesize that such an application will enhance transparency, encourage behavioral changes, increase the use of shared decision-making in clinician-patient communication, improve medication adherence and insight into their own likelihood of relapse and the trajectory of early warning signs. Ultimately, this approach is expected to reduce hospital readmissions, inpatient days, and psychiatric healthcare costs.

## Methods

### Trial design

The planned study (“IT-based relapse monitoring for schizophrenia”; acronym: ILIA) is a randomized-controlled (RCT), multicenter clinical trial evaluating the use of a smartphone- and web-based monitoring system for early warning signs of relapse integrated into standard clinical treatment, compared to treatment as usual (TAU) without digital monitoring. The study is conducted in outpatients with schizophrenia or schizoaffective disorders at 8 psychiatric hospitals in Munich, Augsburg and Berlin (Klinikum rechts der Isar, Ludwig-Maximilians-Universität (LMU) Klinikum, Isar-Amper-Klinikum München-Schwabing, München-Ost, Atriumhaus and Fürstenfeldbruck, Bezirkskrankenhaus Augsburg, Charité Universitätsmedizin Berlin). The inclusion is already possible at the end of an inpatient stay. The RCT is planned for 24 months, with the patients being recruited sequentially for 12 months. The intervention duration itself is 12 months. The protocol is registered in the German Clinical Trials Register (ID: DRKS00034991; registration date: 30.08.2024). An independent ethics committee reviewed and approved the research project (Technical University Munich, approval number: 2024-242-S-KK). The respective ethics committees of the participating clinics have followed the lead opinion of the ethics committee of the Technical University of Munich.

### Participants

We include participants 18 years and older with a diagnosis of schizophrenia or a schizoaffective disorder (ICD-10 F20/F25) who are currently in outpatient treatment. Participants can be at maximum markedly ill (CGI-S and PANSS-6 positive symptoms: delusions, conceptual disorganization and hallucinations ≤ 5) and should have had a relapse within the last two years prior to study inclusion. For this study, we defined relapse as a past hospitalization or a clinical increase in psychotic symptoms. The relapse inclusion criterion does not apply to patients whose initial disease onset occurred less than three years ago. Participants should be able to give their informed consent before the study starts. Participants must not be experiencing an acute exacerbation at the time of study inclusion, based on the judgment of the treating clinician. They further should not have any other cognitive or physical impairments that prevent adequate study participation.

### The smartphone-based monitoring app and the digital clinic portal for feedback

In the ILIA-study we collaborate and license the products of Mindpax.me, a Czech-based company founded in 2015, emerged from the intersection of mental health research and digital innovation, aiming to improve the monitoring and management of mental health disorders through data-driven wearable technologies and advanced analytics. Mindpax.me offers continuous, long-term disease monitoring systems for patients with psychiatric disorders to provide psychoeducational suggestions to increase their competency of self-management, prevent relapse and increase the duration of remissions [31].

#### The early-warning-signs questionnaire (EWSQ-10P)

During the 12-month intervention period, the participants in the intervention group enter ten possible early warning signs according to the EWSQ-10P [27] in an app on their own smartphones on a weekly basis. The EWSQ-10P is a scale used to assess the following early warning signs: Sleep disturbances, loss of appetite, concentration difficulties, increased anxiety, paranoia, inner restlessness/irritability, loss of energy, difficulty solving everyday problems and acoustic hallucinations. The 10th item of the EWSQ-10P is a personalized early warning sign and can be set and changed individually by the patient during the course of the study. The EWSQ-10P assesses the change in symptomatology compared to the previous week, i.e., the last questionnaire. The EWSQ-10P has adequate internal consistency and convergent validity [27]. We build upon the original ITAREPS algorithm for generating alerts for impending relapse (access to more than 600 patient years). If the ITAREPS algorithm, using total score and sub-scores of the current, as well as previous EWSQ-10P exceeds a certain threshold, a warning signal is issued, notifying both, patients and clinicians. Upon the warning, the patient enters an alert state and the EWSQ-10P is requested twice a week. The alert algorithm uses a setting that is more sensitive to further deterioration of the clinical status and increased relapse risk.

#### Functionalities of the sigma.me app

**Fig. 1:**
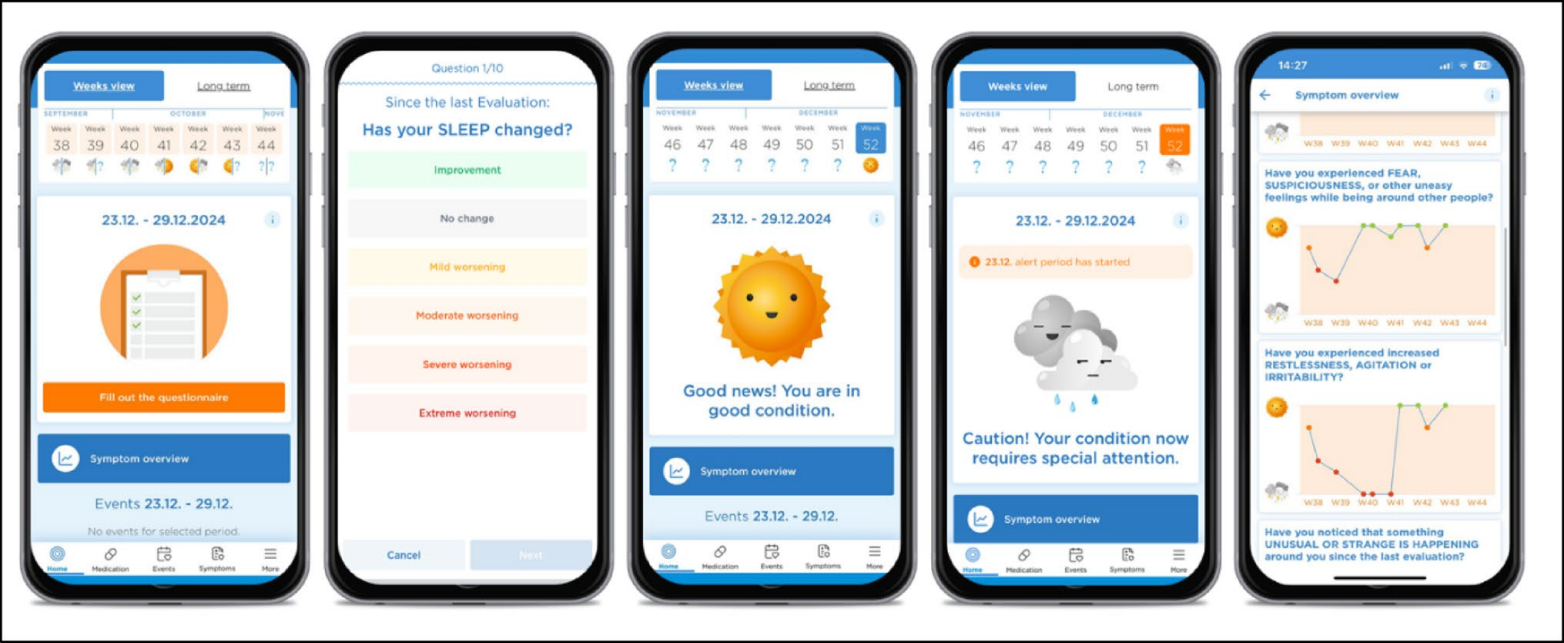
Interface of the Sigma.me App

The ˈSigma.meˈ App runs on iOS and Android systems. Figure 1 shows the interface of the ˈSigma.meˈ App that is used in the intervention group. From left to right, the home screen shows the notification to complete an upcoming EWSQ-10P questionnaire, as well as the health status overview of the past weeks. The second interface of Fig. 1 shows an example question from the EWSQ-10P. Answers are shaded in colour depending on the answer option to ease the filling out procedure for the patients. This design was adopted based on a participatory preliminary study phase, in which patient representatives gave feedback on the user interface in focus groups. The next two interfaces show the graphical feedback on the home screen depending on varying answers in the questionnaire and the respective change in health status. Participants can also insert any change in medication in-take or life events (e.g. challenges at work or within relationships) and mark those events as a trigger. This allows the patient to indicate events that may worsen their condition and avoid them in the future. A long-term overview screen allows the patient to see the course of their relapse risk and quickly recognize the relationships between events, medication changes, and their mental state. Clicking on “symptom overview” directs participants to the very right interface depicted in Fig. 1. Here, patients can inquire about the detailed history of their health, broken down into the ten early warning signs.

**Fig. 2:**
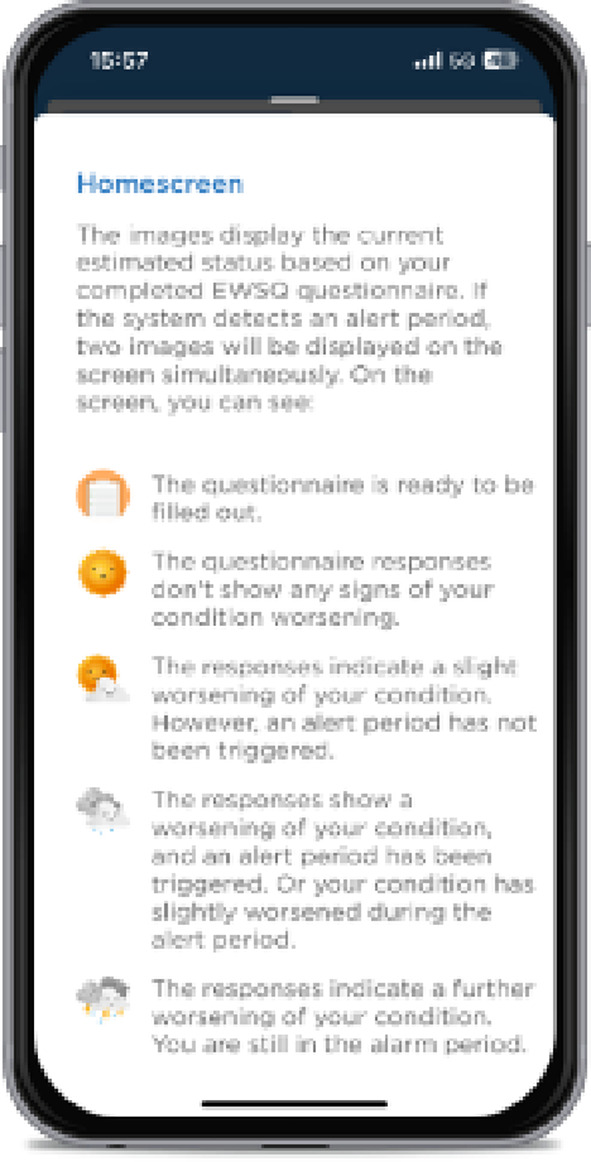
Info-Button of the home screen that explain depiction of health status in the form of weather icons

Graphical depictions of weather icons within the smartphone app represent participants´ current health status based on questionnaire responses (Figs. 1 and 2). The various icons and their meanings are as follows: The smiling sun indicates no deterioration based on the questionnaire responses, suggesting that the patient is stable. The sun with a few clouds reflects a slight deterioration, but not enough to trigger an alarm period. The rain cloud represents either a threshold exceedance, triggering the alarm period, or a slight but not significant deterioration during an ongoing alarm period. The thunderstorm clouds signify a significant deterioration during the current alarm period, prompting the three-week alarm period to restart.

In an alert state, patients are required to complete the EWSQ-10P twice a week for three weeks and an orange shaded banner on the home screen reminds the patient of the alert status. The alert period lasts for a minimum of three weeks. Its extension depends on further deterioration of the patient’s condition. If the patient’s condition does not worsen further, the alert period automatically ends and patients resume completing the questionnaire weekly. The change in status can be seen by a shift in the weather icon from rain to sunshine. The wording and clarity of these icons were participatory reviewed and adjusted in focus groups during the preliminary study phase in collaboration with patient representatives to ensure their comprehensibility and effectiveness.

#### Digital clinic portal for clinicians

**Fig. 3:**
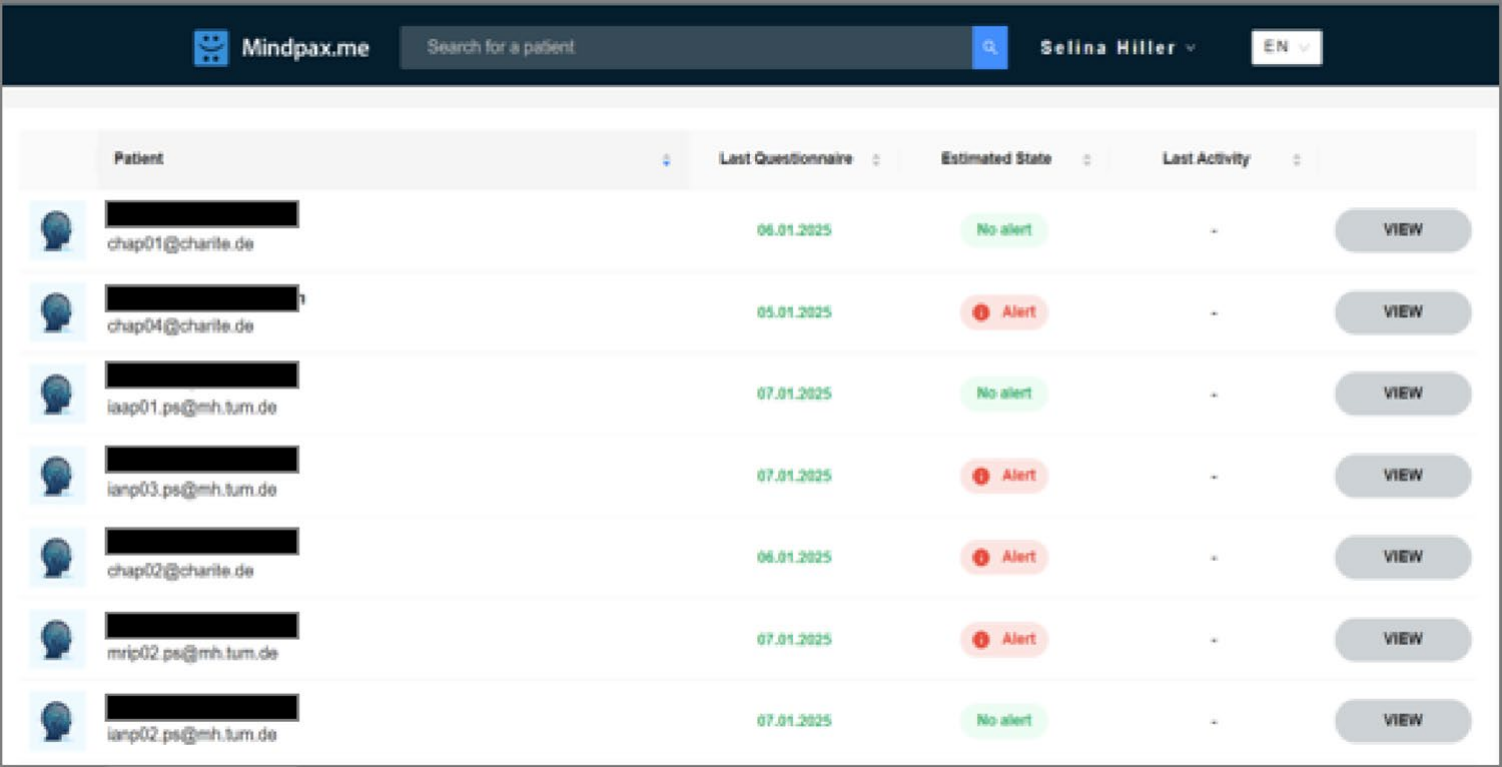
Home screen in the digital clinic portal with patient overview

**Fig. 4:**
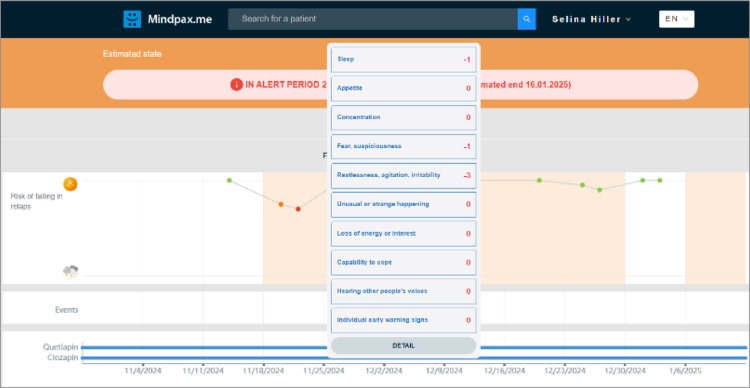
Overview of the digital clinic portal: Single patient status dashboard (overall risk of falling in relapse)

The information from the app is sent to and processed on a secure server. Clinicians can view the course of the early warning signs at any time through access to a digital clinic portal via a web-application. Figure 3 shows the home screen of the digital clinic portal, with an overview of included patients, the date they last completed the ESWQ-10P and their current health status. Clicking on the button “View” takes clinicians to the interface depicted in Fig. 4. Similar to within the app, a dotted line shows the course of relapse risk of the respective patient during the past weeks. The colour of the dots helps to indicate the change in status, i.e., green indicates no worsening. Clicking on the dots takes clinicians to a detailed table showing the changes in the ten early warning signs compared to the previous week.

Clinicians are notified via email in four cases: A patient (1) enters the alert state, (2) extends the alert state, (3) exits the alert state or (4) missed a questionnaire twice in a row. The latter scenario is called “silent patient” and could constitute another early warning sign of relapse. In all cases, except for a patient exiting an alert period, the patient and clinician should promptly get in touch, e.g. via phone, to assess the patientˈ current (mental) health status. This is a shared responsibility of both, the patient and the clinician. As part of a shared decision-making process, the next treatment steps should be decided in a participatory manner. The action to be taken in response to an alert is not prescribed by the study and is left entirely to the shared decision-making between the clinician and the patient. For example, they may decide to provide psychoeducation, to improve sleep hygiene, issue a sick note, possibly adjust antipsychotic medication, or jointly deciding that no action is needed.

After the joint conversation about an (extended) alarm, clinicians must document the measures decided upon. This happens within the digital clinic portal, using a “Notes” function embedded in every patient profile. Additionally, a study team member will review the measures implemented in response to an alert at the next study visit.

### Baseline set-up and technical support for the digital intervention

Before the study starts, clinicians from participating centers undergo multiple introductory sessions, including a final one-on-one on-boarding session, in which the account in the digital clinic portal is set up together study team members. For patients, after baseline assessment and randomization, participants of the intervention group download the Sigma.me app on their smartphone. Study staff assists patients with the set-up and registration procedure. Patients receive a pseudonymized study email as their user name. The study team acts as a first level helpline for patients and clinicians, e.g. in case of lost passwords or problems with the app and portal usage.

Built-in help buttons assist patients to navigate the app, when no assistance is available. Help-buttons explain the weather icons (Fig. 2), how the EWSQ-10P questionnaire assesses changes compared to the previous week, and how to add new symptoms or change the personalized early warning sign.

Moreover, printed fact sheets are handed to patients and clinicians (see Multimedia Appendix 1). Patients can refer to these sheets to review their log-in details, while clinicians can use them to review the study procedure in case of an alert period. These fact sheets serve as quick reference guides for navigating the respective interfaces and understanding their functionalities. The fact sheets also provide contact details for technical support, ensuring that patients and clinicians can contact the study team at any time and have the resources to use the applications effectively. A regular newsletter helps to keep participating clinicians up-to-date and bimonthly video conference calls are offered to clarify open questions, help with technical issues or share experiences on implementation barriers or best-practice examples.

### Sample size and recruitment

We aim for a sample size of *N* = 110 patients recruited from *n* = 8 clinics. The sample size calculation is based on the outcome “number of patients requiring inpatient psychiatric admission within one year.” A randomized pilot study using a simple text message-based solution to monitor early warning signs showed large effect sizes [28]. Since we expect the pilot study effect to be inflated and cluster effects present in our study, we opt for a considerable smaller, but still moderate effect (w = 0.30) for sample size calculation. Using a chi-squared test without cluster effects, a group size of 44 patients per group provides a power of 80% to detect a population effect of w = 0.30 with a two-sided Type I error rate of 5%. Assuming a dropout rate of approximately 20% [28], 110 patients need to be recruited. An approximately equal distribution across the eight centers is planned.

Identification of suitable patients and recruitment is supported by local clinicians in the clinics. Flyers and posters at local study sites support the recruiting process. Additionally, patients are recruited at external centers, such as counseling centers, assisted living groups, or self-help groups. To ensure a comprehensive recruitment process, social media is also included. Experienced study staff at the lead center, Klinikum rechts der Isar in Munich, and the Berlin study center are responsible for enrolling participants in the study, including obtaining informed consent. External patients are getting affiliated with one of the participating clinics for the duration of the study. They can choose to fully transfer to our outpatient clinics during the study period or remain with their local doctor while simultaneously attending the participating outpatient clinic.

A study phone will be provided for patients who do not own a private smartphone but still possess basic digital skills, such as using a PC or tablet, and having an internet connection at home. However, these cases are reviewed individually and thoroughly to ensure that no bias arises.

### Randomization and blinding

**Fig. 5:**
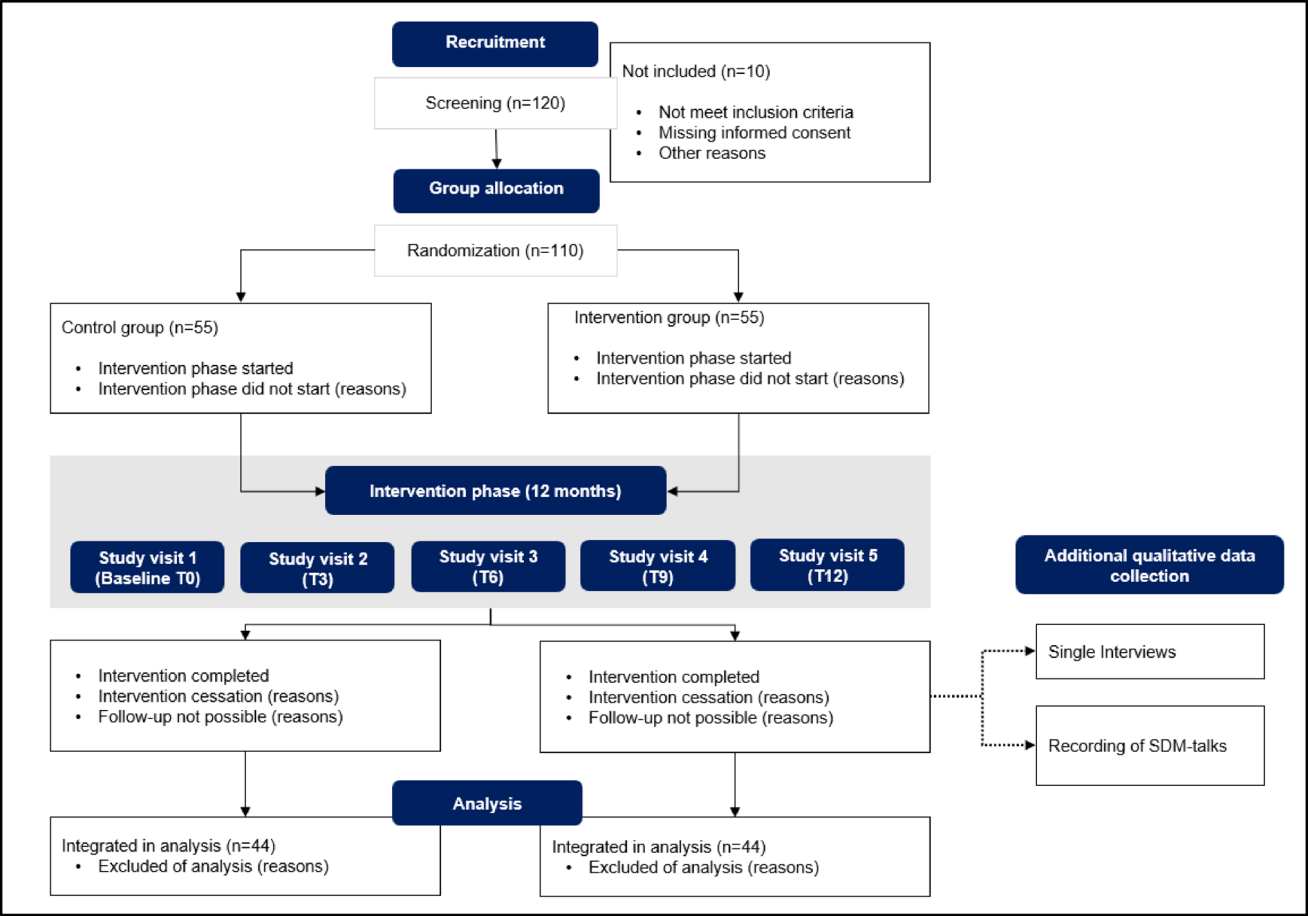
Study flow diagram. Randomization before T0. Five study visits every 3 months for 12-month study duration. Qualitative data collection with a representative subsample of study participants

Individual randomization (1:1) ensures a balanced distribution of participants and their treating clinicians between the intervention and control groups across all centers (Fig. 5). Randomization is automatically conducted after obtaining informed consent and prior to the baseline assessment using the randomization tool in RedCap, a secure web-based electronic data capture tool hosted at Yale University, designed for data collection and management in research studies [32, 33]. Due to the nature of the intervention, blinding is not possible.

### Outcomes

#### Baseline parameters

At baseline (T0), sociodemographic data (age, sex, nationality and language, educational background, family and occupation status) and history of illness (ICD-10 diagnoses, duration of illness, number of previous hospitalizations, previously prescribed medications, substance abuse) is collected via patient and clinician-rated questionnaires (Table 1).

**Table 1:** Planned timepoints and data collection

Scale/item	Visit 0 (T0)	Visit 1 (T3)	Visit 2 (T6)	Visit 3 (T9)	Visit 4 (T12)
	Baseline	Week 13 +/- 3 weeks	Week 26 +/- 3 weeks	Week 39 +/- 3 weeks	Week 52 +/- 3 weeks
Written informed consent^a^	X^b^				
Screening/Assessment of inclusion/exclusion criteria^c^	✓^d^				
Sociodemographic characteristics	X				
Psychiatric diagnose(s) after ICD plus code	✓				
Change of psychiatric diagnosis(es)		✓	✓	✓	✓
Duration since first diagnosis	✓				
Number of previous episodes until now	✓				
Psychiatric comorbidities	✓				
Number of hospitalizations until now	✓				
Hospitalization days due to psychiatric illness in the past two years	✓				
Substance abuse	X	X	X	X	X
Alcohol abuse	X				
Smoking	X				
Currently psychiatric medication(s)	✓	✓	✓	✓	✓
Change and date in psychiatric medication(s)		✓	✓	✓	✓
Current other measures: e.g., psychotherapy, group therapy	✓	✓	✓	✓	✓
Rehospitalization (yes/no) and brief summary of admission reasons		✓	✓	✓	✓
Days in hospital since last study visit		✓	✓	✓	✓
Time to hospitalization		✓	✓	✓	✓
eHeals-g	X				
Questionnaire on prospective acceptance of eHealth	X and ✓				
Alarms since last study visit		✓	✓	✓	✓
Measures taken, if any, following the alarm		✓	✓	✓	✓
EWSQ-10P (control group only)	X	X	X	X	X
SDMQ-9	X	X	X	X	X
PAM13	X	X	X	X	X
ZUF-8	X	X	X	X	X
15-STARS	X	X	X	X	X
Question on Adherence	X	X	X	X	X
CGI-S/C	✓	✓	✓	✓	✓
PANSS-6	✓	✓	✓	✓	✓
FORSE	X	X	X	X	X
EuroHisQoL	X	X	X	X	X
WHODAS 2.0	X		X		X
SOFAS	✓	✓	✓	✓	✓
ANTISIDES	X				X
UKU-SERS self-rating	X				X
Mannheimer Resource Consumption Module	X				X
Reason for Dropout^e, f^ (if applicable)		✓	✓	✓	✓

^a^Written informed consent must be obtained at Baseline Visit 0 before data collection starts

^b^A X indicates the scale is patient-rated

^c^Screening can be done at Visit − 1 before Baseline or on the same day of Baseline at Visit 0

^d^A ✓ indicates the scale is clinician-rated

^e^Reasons for drop-out: Ineffectiveness of treatment, deterioration of condition, medication side effects, non-compliance, patient withdrawal of consent, lost-to-follow-up, additional adverse event, others

^f^In case of a drop-out, an early-termination visit should be conducted like the planned end-of-study visit (Visit 4, week 52). For qualitative interviews, the same procedure applies

To assess relevant factors contributing to the adherence to the digital intervention and its efficacy, we ask patients and clinicians about their acceptance of digital health interventions [34]. Patients additionally provide answers to the German version of the eHeals questionnaires (eHeals-g) on health-related Internet use [35].

#### Primary outcome

As the primary outcome we evaluate whether the 12-month intervention phase, during which the intervention group utilizes a smartphone-based relapse monitoring app that monitors early warning signs in addition to treatment as usual (TAU), reduces the number of patients readmitted to the hospital due to psychiatric reasons at least once during the study period. Hospitalization is defined as an increased level of care, including an inpatient stay of at least one day, a day patient stay, or home treatment due to exacerbation of psychiatric symptoms (not social reasons). Therefore, hospitalizations (if applicable) are assessed at every study visit, i.e., every three months, following the baseline assessment (T0).

#### Secondary outcomes

To complement our primary outcome of hospitalizations, we assess whether the intervention reduces the days spent in hospital and the time to hospitalization at three month-interval study visits (T0, T3, T6, T9, T12).

At every study visit (T0 – T12) we assess whether the app promotes more shared decision-making in the course of treatment (Shared Decision Making Questionnaire, SDM-Q9) [36], changes fear of relapse (Fear of Recurrence Scale Questionnaire, FORSE) [15], increases patient satisfaction (Patient Satisfaction Questionnaire, ZUF-8) [37] and patient activation (Patient Activation Measure, PAM-13) [38], and thus potentially improving medication adherence (Screening Tool for AdheRence to medicineS, 15-STARS) [39].

To better understand whether the intervention improves symptomatology, we use the 6-item version of the Positive and Negative Syndrome Scale (PANSS-6) [40] and the Clinical Global Impression Severity/Improvement (CGI-S/C) [41]. All three scales are rated by the clinician after patient study visit (T0 – T12). The clinician-rated Social and Occupational Functioning Assessment Scale (SOFAS) [42] is also assessed at the same timepoints.

Changes in Quality of Live (QoL) are assessed via the patient-rated EUROHIS-QOL [43] every three months. Patients report changes in social/global functioning via the WHODAS 12-item scale [44] at T0, T6 and T12. We evaluate the presence and severity of medication side effects using the Udvalg for kliniske undersøgelser scale (UKU-SERS) [45] and patient-reported antipsychotic and antidepressant side-effect scale (ANTISIDES). Those two measures are assessed at baseline (T0) and after study completion (T12).

To evaluate whether the implementation of a smartphone-based relapse monitoring smartphone is cost-effective from a pharma-economical perspective, we use the Mannheimer Resource Consumption Module Questionnaire at T0 and T12 [46].

#### Qualitative data

A qualitative approach complements the quantitative data collection, enhancing result validity and facilitating interpretation. Using a semi-structured interview guide, built upon previous clinical intervention studies conducted by our research group in this target population [47–49], questions regarding the feasibility, implementation, practicality, fear or relapse, potential side effects, and potential effects of the proposed digital intervention is addressed in single interviews with participants of the intervention group at the end of the study phase. We conduct approximately 20 interviews until content saturation is reached [50]. Additionally, we record sessions between clinicians and patients (i.e., approximately 20 patient-clinician dyads; until content saturation is reached) during the study period in both conditions, i.e., intervention and control group, to observe direct interactions during treatment, effects of the proposed intervention on shared-decision making, utilization of the digital intervention and potential differences in the groups in terms of conversation analysis.

### Data analysis

#### Analysis of quantitative data

The primary outcome parameter (psychiatric hospital admission) will be analyzed using a mixed model (logistic regression) with random intercepts for wards. Exploratory analyses of secondary outcome parameters will also be conducted: Differences in the time to hospitalization and simple relapses will be analyzed using a Kaplan-Meier curve and the log-rank test or within a generalized linear model framework (“Piecewise Exponential Model”). Additionally, we want to explore whether the effect of treatment is related with eHealth literacy and the acceptance of digital interventions with a subgroup analysis. Other secondary outcome parameters will be analyzed using mixed-effect regressions with random intercept for wards (logistic regressions if binary and linear regressions if continuous). If there are repeated measures available, this will be investigated within a multilevel framework, with random intercepts for subject and random slopes for time, if applicable.

Following the ITT principle, people that withdraw from the study (missing follow-up) need to be accounted for in the analysis. To deal with missingness, the primary analysis will adjust for baseline PANSS-6 score and age, since we expect these variables to be related to missingness. We expect an adjusted analysis to be the most efficient way to account for missing data in our experiment, mostly because of the binary outcome and the structure of missingness that is common in RCTs [51, 52]. The considerations for missing data will be subject to a sensitivity analysis. For this, missing values will be imputed with MICE (Multiple Imputation for Chained Equations) under a Missing at Random assumption. Imputation is an attractive alternative to our primary analysis, since there are many possible auxiliary variables measured at baseline that are not incorporated into the adjusted primary analysis. Additionally, in the case that there is a lot of dropout of similar structure (Reasons for Dropout), a sensitivity analysis under a MNAR assumption (pattern-mixture models) will be conducted.

#### Analysis of qualitative data

For the qualitative approach, semi-structured interviews will be used to undergo a process evaluation analysis, to evaluate and explain the outcomes [53, 54]. The interview protocol will be adapted by previous protocols of clinical intervention studies conducted by the research group in this target population [47–49]. We will take recordings of patient-clinician dyads for the purpose of conducting conversation analyses based on conventions developed by Gail Jefferson [55], to observe direct interactions during treatment and utilization of the digital intervention. Basic and fine-grained transcripts will be prepared for selected relevant sections to examine the conversations with regard to the themes of shared decision making, fear of relapse and treatment adherence. The dyadic records are also analysed in a structured content analysis [56]. This approach allows for fully inductive categorization of the material, deductive categorization, and a combination of both through a multi-stage process and coding.

## Discussion

Digital interventions have emerged as promising tools for supporting patients with schizophrenia or related psychotic spectrum disorders, especially in the critical post-hospital discharge phase [57–59]. These interventions encompass a wide range of applications, from symptom monitoring and relapse prevention to cognitive training and psychosocial support [60–62]. Digital tools can provide ecologically valid data for clinicians, contributing to more accurate, informed decisions and better personalized and proactive care [63]. These low-threshold interventions can empower patients to take an active role in their treatment and be more involved, thereby enable more autonomous health actions [64].

Interview data reveals that patients with schizophrenia often overestimate their health status and underestimate their risk of relapse [65]. This tendency stems from a limited ability to recognize early warning signs, leading to maladaptive behaviors during critical phases. Given the high relapse rate in schizophrenia or schizoaffective disorder and associated costs, those results underscore the need for improved risk awareness and timely, accurate communication between patients with schizophrenia and clinicians, to manage relapse risk effectively [65]. By providing comprehensive symptom monitoring and fostering patient autonomy, digital interventions can contribute to more effective and timely relapse prevention strategies. A pilot RCT showed that text message-based relapse prevention measures are highly acceptable and easy to understand for patients with schizophrenia or a schizoaffective disorder and can improve patient-clinician communication [66].

While research on blended care with digital solutions for patients with schizophrenia is expanding, the findings remain tentative at best, and the widespread integration of tailored digital tools at a national level is still largely absent [67]. Previous text message-based early warning sign monitoring solutions followed a paternalistic medical approach and focused solely on the actions of the clinicians, excluding patients from the information loop and no visual feedback on the disease development was provided to neither the patients, nor clinicians [28, 66, 68]. Accordingly, the effects on reduced hospitalizations depended mainly on the behavior of the clinicians, whether and how quickly they reacted to the text message-based relapse alert [28, 69, 70]. To our knowledge, only one cluster-randomized trial exists that used a smartphone-based intervention to prevent relapse in people with schizophrenia by monitoring early warning signs [71, 72]. Their smartphone app required daily monitoring with up to 56 items per assessment, delivered empowerment impulses to participants and assistance provided by peer-support workers. However, to our knowledge, no validated questionnaire was included, the intervention did not provide a digital interface and feedback loop to the treating clinician, and no joint action between the patient and the treating clinician was mandatory. Additionally, the number of assessments during the 12-month intervention period appears to be quite burdensome. The study did not focus on the assessment of hospitalization measures (e.g. days spent in hospital, time to hospitalization) as main indicators of effectiveness on relapse.

With our study we expect that the continuous digital monitoring of early warning signs by patients and the participatory treatment decision-making process together with their clinicians, will lead to behavioral changes, thereby reducing the necessity and duration of psychiatric hospital stays of patients, as well as healthcare costs. Furthermore, the intervention could improve symptomology, medication adherence, fear of relapse, satisfaction, social functioning, patient activation and shared-decision making. The insights gained from this study can be applied to routine psychiatric care, sustainably enhancing the treatment of patients with schizophrenia and schizoaffective disorder.

## Electronic Supplementary Material

Below is the link to the electronic supplementary material.

Multimedia Appendix 1

## Abbreviations

RCT: Randomized-controlled trial
TAU: Treatment as usual
EWSQ-10P: Early-Warning-Signs Questionnaire – 10 items, Patient Version
T0: Baseline Assessment; First Study Visit
MICE: Multiple Imputation with Chained Equations
