# Supplementary material for: The ILIA study: protocol for a randomized-controlled multicenter clinical trial on smartphone- and web-based relapse monitoring for patients with schizophrenia or schizoaffective disorder
Source: Eur Arch Psychiatry Clin Neurosci. 2025 Aug 19;276(2):637–50. doi: 10.1007/s00406-025-02089-7 (PMC12953440; doi:10.1007/s00406-025-02089-7)
Supplement: Supplementary file 1 — Multimedia Appendix 1 [file 406_2025_2089_MOESM1_ESM.pdf]

Bei Fragen oder techn. Problemen:
E-Mail an selina.hiller@tum.de
Telefon 089 4140 6430

ILIA-Studie – Merkblatt für ÄrztInnen und PsychologInnen

Virtuelles Klinikportal:
https://sigma.mindpax.me/

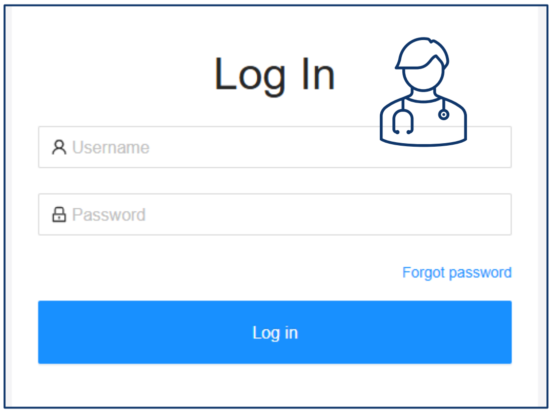

Username: Ihre E-Mail
Passwort: Wie angegeben bei der Registrierung in der App

Sie können den Fragebogen ausfüllen.

Die Antworten auf den Fragebogen zeigen keine Anzeichen für eine Verschlimmerung Ihres Zustands.

Die Antworten deuten auf eine leichte Verschlechterung Ihres Zustands hin. Eine Alarmperiode wurde jedoch nicht ausgelöst.

Die Antworten zeigen eine Verschlechterung Ihres Zustands an, und es wurde eine Alarmperiode ausgelöst. Oder Ihr Zustand hat sich während der Alarmperiode leicht verschlechtert.

Die Antworten deuten auf eine weitere Verschlimmerung Ihres Zustands hin. Sie befinden sich daher weiter in der Alarmperiode.

Die Antworten zeigen, dass sich Ihr Zustand nicht weiter verschlechtert oder verbessert hat.

? Ihr Zustand ist unbekannt. Der Fragebogen wurde (noch) nicht ausgefüllt.

In folgenden 4 Fällen werden Sie via E-Mail benachrichtigt:

1. Basierend auf den Antworten im Fragebogen, befindet sich ein/e PatientIn in der **Alarmperiode**
2. Basierend auf den Antworten im Fragebogen, befindet sich ein/e PatientIn **weiterhin** für einer Woche in der **Alarmperiode** (weitere Verschlechterung)
3. Ein/e PatientIn hat den Fragebogen nicht ausgefüllt („**silent patient**“)
4. Ein/e PatientIn hat die **Alarmperiode verlassen**

Bitte beachten Sie: Sie erhalten diese E-Mails für jeden Patienten und jede Patientin, die in Ihrer Ambulanz eingeschlossenen werden.

- Im Online-Portal wird nur das Studienpseudonym oder der Benutzername angezeigt
- Daher müssen Sie eine **Pseudonymisierungsliste** mit all **Ihren PatientInnen** führen, die für die Studie eingeschlossen werden. So wissen Sie, um welche/n PatientIn es sich handelt

BITTE WENDEN

ILIA-Studie – Merkblatt für ÄrztInnen und PsychologInnen

Was muss während der Alarmperiode passieren?

In **Pseudonymisierungsliste** nachsehen, um welche/n PatientIn es sich handelt (anhand angezeigter Studien E-Mail / Benutzername im Dashboard)

So schnell wie möglich Kontaktaufnahme mit PatientIn zur Besprechung potentieller Maßnahmen

Wichtige Anmerkung: Der Beginn einer Alarmperiode soll bei dem/der Patient/in **keinesfalls Angst auslösen**. Bitte vermitteln Sie Ihrem/Ihrer Patient/in, dass es sich um eine **Präventionsmaßnahme** handelt und nun dem Gesundheitszustand **besondere Aufmerksamkeit** geschenkt wird.

Beschluss/Maßnahme des Shared-Decision Making **im Klinikportal dokumentieren**

Der Beschluss / die Maßnahme wird anhand der **„Verlaufsdokumentation“-Funktion** eingegeben. Durch klicken auf „Neuen Eintrag hinzufügen“ können Sie mit Hilfe einer Notizfunktion und eines Drop-Down Menüs die jeweilig zutreffende Maßnahme auswählen. Bitte dokumentieren Sie auch Datum und Art der Kontaktaufnahme.

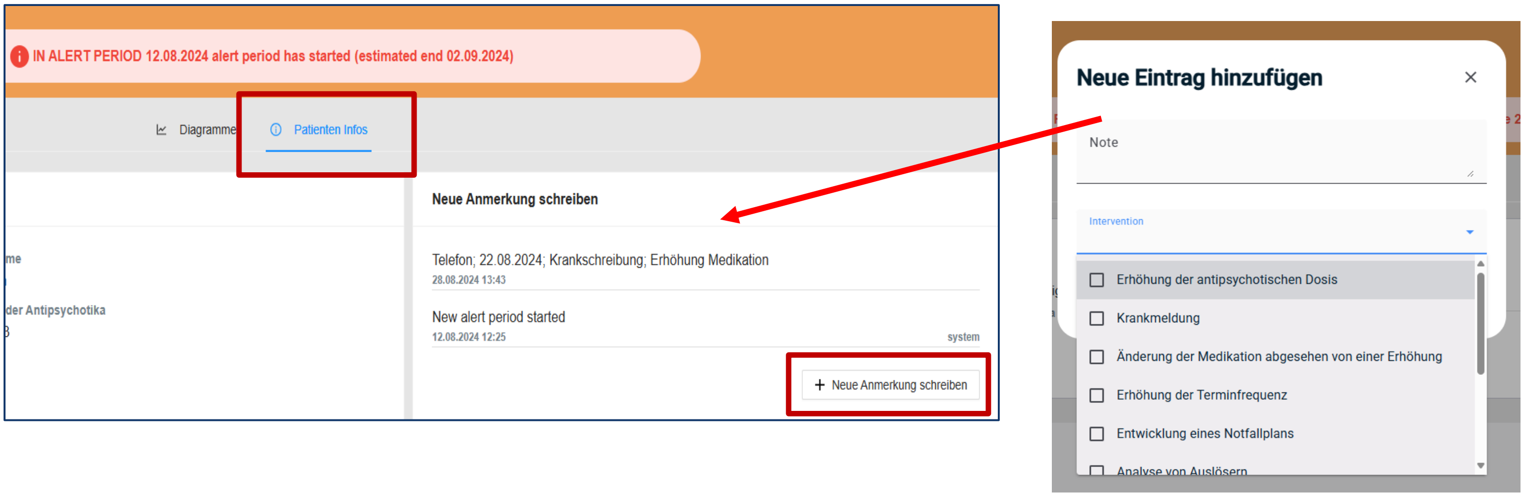

Notizen

Merkblatt für Teilnehmende der ILIA-Studie

Liebe Studienteilnehmerin, Lieber Studienteilnehmer,

Auf diesem Merkblatt können Sie Ihre individuellen Daten zum einloggen in die App vermerken. Bei Fragen oder Problemen können Sie sich jederzeit an uns wenden:

Unsere Kontaktdaten: Telefon 089 4140 6430 & E-Mail: selina.hiller@tum.de

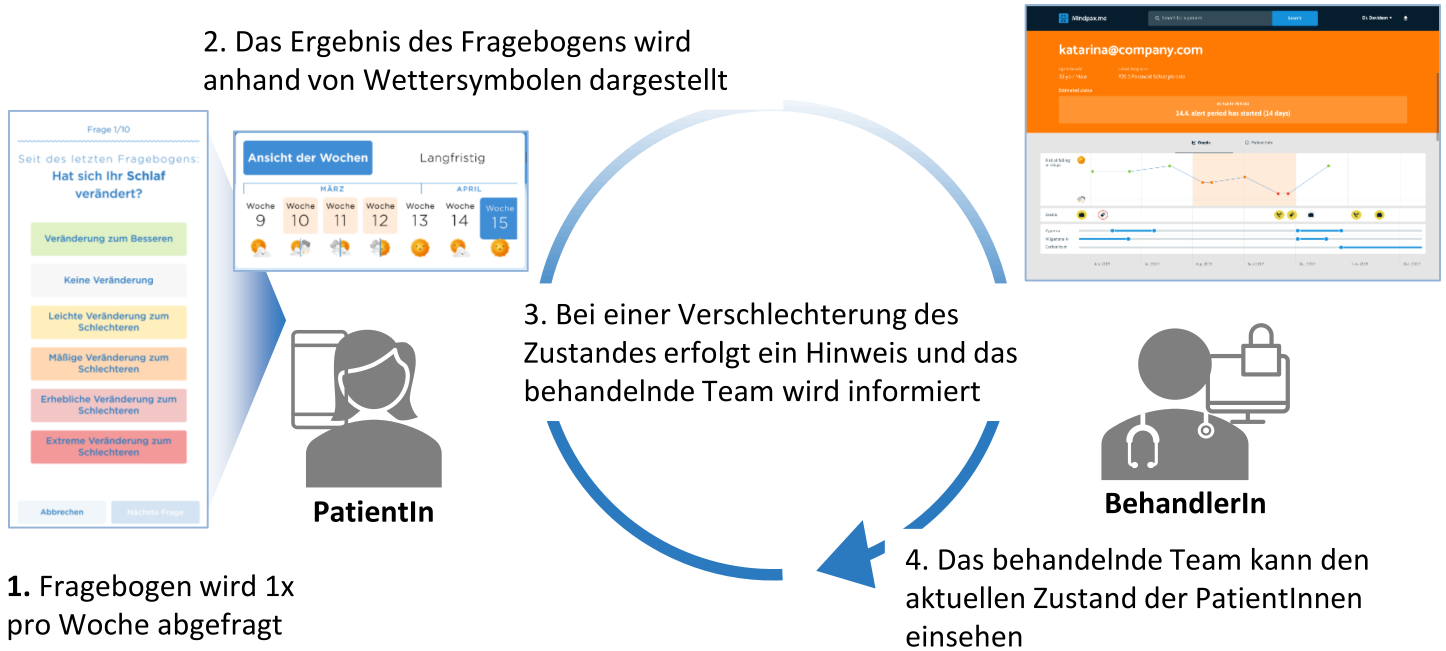

Für den LOG – IN

Studien E-Mail:

Benutzername:

(z.B. Anfangsbuchstaben, Nickname, etc.)

Passwort:

PIN-Code:

Solange Sie sich nicht aus der App ausloggen, ist beim Öffnen der App **nur Ihr PIN-Code** erforderlich

Sobald Sie sich **ausloggen**, müssen Sie sich beim nächsten Mal wieder mit Ihrer **Studien E-Mail/ Benutzername und Passwort** anmelden

Wenn Sie Ihren **PIN vergessen**, können Sie sich **einfach ausloggen und wieder einloggen** und einen **neuen PIN** vergeben
